# Comparing Ozonation and Biofiltration Treatment of Source Water with High Cyanobacteria-Derived Organic Matter: The Case of a Water Treatment Plant Followed by a Small-Scale Water Distribution System

**DOI:** 10.3390/ijerph15122633

**Published:** 2018-11-24

**Authors:** I-Chieh Chien, Sheng-Pei Wu, Hsien-Chun Ke, Shang-Lien Lo, Hsin-hsin Tung

**Affiliations:** 1 Department of Water Resources and Environmental Engineering, Tamkang University, 151 Yingzhuan Road, Tamsui District, New Taipei City 25137, Taiwan; icchien@mail.tku.edu.tw; 2 Graduate Institute of Environmental Engineering, National Taiwan University, No. 1, Sec. 4, Roosevelt Rd., Taipei 10617, Taiwan; nostril23@gmail.com (S.-P.W.); f54971257@gmail.com (H.-C.K.); sllo@ntu.edu.tw (S.-L.L.)

**Keywords:** cyanobacteria bloom, disinfection byproducts, biofiltration, ozonation

## Abstract

High cyanobacteria-derived dissolved organic carbon (DOC) in source water can cause drinking water quality to deteriorate, producing bad taste, odor, toxins, and possibly elevated levels of disinfection byproduct (DBP) precursors. Conventional water treatment processes do not effectively remove algal organic substances. In this study, rapid-sand-filtration effluent from a water treatment plant on Kinmen Island, where serious cyanobacterial blooms occurred, was used to evaluate the DOC- and DBP-removal efficiency of ozonation and/or biofiltration. To simulate a small-scale water distribution system following water treatment, 24 h simulated distribution system (SDS) tests were conducted. The following DBPs were analyzed: trihalomethanes (THMs), haloacetic acids (HAAs), haloacetonitriles (HANs), and trichloronitromethane (TCNM). Applying biological activated-carbon filtration (BAC) on its own achieved the greatest reduction in SDS-DBPs. Ozonation alone caused adverse effects by promoting THM, HAA, and TCNM formation. Ozonation and BAC filtration yielded better DOC removal (51%) than BAC filtration alone (41%). Considering the cost of ozonation, we suggest that when treating high cyanobacterial organic matter in water destined for a small-scale water distribution system, BAC biofiltration alone could be an efficient, economical option for reducing DBP precursors. If DOC removal needs to be improved, preceding ozonation could be incorporated.

## 1. Introduction

Cyanobacterial blooms in source water can impact tap water quality and pose a serious health threat. Not only can cyanobacteria produce various toxins [1,2] and compounds responsible for taste and odor problems [3,4], they can also generate algal organic matter (AOM), which has been identified as a precursor of various disinfection byproducts (DBPs) when chlorinated or chloraminated in water treatment processes [5,6,7,8]. Increases in the frequency and intensity of cyanobacteria blooms in source water have been linked to human population growth, climate change, and extreme weather events, implying that the treatment of water with excess cyanobacteria growth may become a serious challenge for water treatment facilities in the future.

The processing by conventional means of cyanobacteria-derived organic substances, including both intracellular and extracellular organic matter (IOM and EOM respectively), has been found to be subject to limitations. For example, the generation of complexes between algal organic matter and coagulants may inhibit coagulation. Cellular organic matter (such as peptides and proteins) produced by the cyanobacterium *Microcystis aeruginosa* can increase the quantity of coagulants required [9,10,11,12]. Moreover, only a slight reduction in cyanotoxins was reported after conventional flocculation, sedimentation, filtration, and chlorination procedures [13,14]. These findings have led to a recent evaluation of the efficiency of removing AOM by alternative treatment methods.

While ozonation is an increasingly common practice in water treatment plants, the effect of ozone on the reduction of DBPs is a controversial issue. Its impact on the formation of DBP precursors depends on the characteristics of the dissolved organic carbon (DOC) in the source water. Generally, for source water with high concentrations of humics or specific ultraviolet absorbance (SUVA) values (>2 L/mg/m), ozone can reduce the potential for DBP formation by converting the organic matter from more chlorine-reactive, hydrophobic to less chlorine-reactive, hydrophilic fractions. In contrast, ozonation of AOM with low SUVA values typically leads to higher carbonaceous DBP (C-DBP) and nitrogenous DBP (N-DBP) production after subsequent chlorination or chloramination [8,15]. It is therefore important to evaluate the effect of ozonation in terms of DOC and DBP reduction before it is used to treat algae-rich water (i.e., with low SUVA values) [15].

Biofiltration has garnered increasing attention among water facility operators as a means of improving the removal efficiency of DOC and DBP precursors in water treatment processes, particularly for source water containing high concentrations of cyanobacteria-derived organic matter. Wert and Rosario-Ortiz [8] found that most IOM (>99%) extracted from the cyanobacteria *Microcystis aeruginosa*, *Oscillatoria* sp., and *Lyngbya* sp. was biodegradable. Biofiltration is also a suitable process to apply following the application of ozone, since increased biodegradable DOC fractions [16,17] and the presence of biodegradable byproducts such as aldehydes and short-chain carboxylic acids [18] have been reported after ozonation.

The quality of finished water in distribution systems is subject to change before it is delivered to end users, and the degradation level depends on the time or distance involved in transportation (i.e., the size of the distribution system). For example, DOC remaining in finished water in a large-scale water distribution system has more opportunity to react with residual chlorine and could thus form more DPBs. In such systems the assimilable organic carbon in DOC can also increase the risk of bacterial regrowth as more residual disinfectants are consumed. In small-scale water distribution systems, residual DOC in finished water may not be a serious concern, because the relatively high concentration of free chlorine in pipes is capable of inhibiting bacterial activity. Considerations regarding the selection of water treatment processes should therefore take the scale of the subsequent distribution system into account.

The main objective of this study was to assess the feasibility of using ozonation, biofiltration (silica sand or activated carbon as filter media), or a combination of both in a water treatment plant connected to a small-scale water distribution system to treat source water with a high content of cyanobacteria-derived DOC. The yield of trihalomethanes (THMs), haloacetic acids (HAAs), haloacetonitriles (HANs), and trichloronitromethane (TCNM) after each treatment was predicted using 24 h simulated distribution system (SDS) tests to reflect the small-scale water distribution system. The primary research question of this study was: is the replacement of slow sand filtration with ozonation and/or biofiltration, in order to reduce SDS-DBPs, feasible? The results of this study may provide information on how to reduce the DBP yields of water treatment plants that are followed by a small-scale water distribution system, particularly when there have been extensive cyanobacterial blooms in the source water.

## 2. Materials and Methods

### 2.1. Source Water and the Water Treatment Process

**Figure 1:**
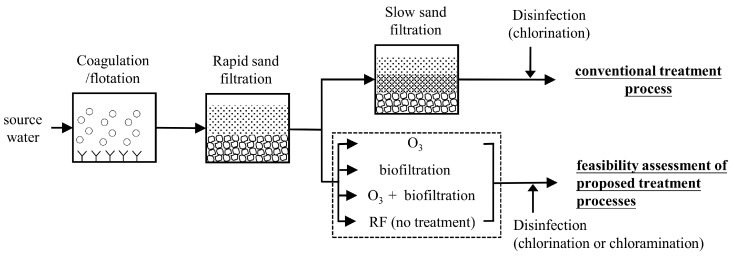
The conventional processes implemented by a water treatment plant on Kinmen Island and the proposed alternative treatments (dashed-line box) of this study. RF—rapid sand effluent.

Effluent from a local rapid-sand-filtration water treatment plant on Kinmen Island was collected on September 22th, 2014 and taken to our laboratory. It was stored at 4 °C before use. Typical source water characteristics were: DOC 12–15 mg C/L, pH 7.16, turbidity 17.43 NTU, conductivity 355.5 μS/cm, dissolved organic nitrogen 0.79 mg/L as N, SUVA 1.53–3.63 L/mg cm [19,20]. Cyanophyta were dominant in the source water (>90%), with an average cell concentration of 7.4 × 10^5^ cells/mL (unpublished data). The treatment process employed at the water treatment plant is illustrated in Figure 1. It consists of coagulation/flotation, rapid sand filtration, slow sand filtration, and disinfection.

### 2.2. Ozonation and Biofiltration

Ozonation was performed by treating water samples with saturated ozone stock solution overnight at 25 °C. Stock solutions were prepared by aerating ozone gas in Milli-Q water (Millipore, Burlington, MA, USA) incubated in a water bath (4–6 °C) for ~30 min. Ozone gas was generated by passing high purity of compressed oxygen gas through ozone generator (Ozonia CFS-2g, Ozonia, Paris, France). Concentrations of ozone in stock solution were measured using the indigo method [21] and were in the range of 0.6–1.0 mM. Final ozone dose was determined using the ratio: 1 mg O_3_ per mg of DOC.

Biofiltration was conducted by passing the water through glass columns packed with either silica sand (hereafter, “Silica”) or activated carbon (hereafter, “BAC”). The column length and inner diameter were 40 cm and 5 cm, respectively. Particles derived from mesh 18 (1 mm) and mesh 20 (0.85 mm) sieves were mixed in equal proportions and used as packing media. A layer of frosted-glass beads was placed at the bottom of the columns to support the filter media. Biofilms on the packing material were pre-matured by feeding both filters with local pond water (DOC 3–6 mg C/L) for at least six months before the experiments began. The empty bed contact time (EBCT) was 20 min. Immediately before the start of the experiments and the collection of biofiltration samples for analysis, 3 L of rapid sand effluent was used to wash the columns.

### 2.3. Simulated Distribution System (SDS) Test

To simulate the short distribution system on Kinmen Island, the residence time was set as 24 h for the SDS tests. The doses of chlorine and chloramine in the SDS tests were set so as to ensure that the free chlorine residuals would be in the range 0.2–1 mg Cl_2_/L after 24 h (Table 1). The chlorine dosage was set so as to meet the regulatory limit of residual free chlorine (0.2–1.0 mg Cl_2_/L) in the water distribution system (Table 1). Although there is no guideline for the chloramine concentration of finished water, the values were set to fall within the same range as for chlorination. The water samples dosed with disinfectants were sealed in amber glass bottles without headspace and stored at 25 °C in the dark. The initial and final concentrations of total and free chlorine were determined using the N,N-diethyl-p-phenylenediamine colorimetric method (Hach method 8167).Untreated rapid sand effluent (RF) was used as a control to evaluate the efficiency of DBP precursor removal by the ozonation and/or biofiltration processes. The treatment processes tested in this study were ozonation (O_3_), silica sand filtration (Silica), biological activated-carbon filtration (BAC), ozonation combined with silica sand filtration (O_3_–Silica), and ozonation combined with BAC filtration (O_3_–BAC).

### 2.4. Analytical Methods

#### 2.4.1. Dissolved Organic Carbon (DOC)

DOC concentrations were measured in a wet oxidation TOC analyzer (Aurora 1030W TOC Analyzer, OI Analytical, College Station, TX, USA) according to the manufacturer’s protocol. Briefly, 5% phosphoric acid (85%, Merck, Darmstadt, Germany) was used to convert inorganic compounds to carbon dioxide, which was then purged out of the liquid phase using nitrogen gas. A 10% sodium peroxydisulfate solution (≥99%, Sigma-Aldrich, St. Louis, MO, USA) was used to oxidize residual organic compounds to form carbon dioxide. The concentration of carbon dioxide produced was measured by a non-dispersive infrared (NDIR) detector. All samples were pre-filtered through a 0.45 μm membrane before the DOC analysis.

#### 2.4.2. Disinfection Byproducts (DBPs)

The following C-DBPs and N-DBPs were measured in this study: (1) four trihalomethanes [THM_4_; the sum of chloroform (TCM), bromodichloromethane (BDCM), dibromochloromethane (DBCM), and bromoform (TBM)], (2) nine haloacetic acids [HAA_9_; the sum of monochloroacetic acid (MCAA), monobromoacetic acid (MBAA), dichloroacetic acid (DCAA), trichloroacetic acid (TCAA), bromochloroacetic acid (BCAA), dibromoacetic acid (DBAA), bromodichloroacetic acid (BDCAA), chlorodibromoacetic acid (CDBAA), and tribromoacetic acid (TBAA)], (3) four haloacetonitriles [HAN_4_; the sum of trichloroacetonitrile (TCAN), dichloroacetonitrile (DCAN), bromochloroacetonitrile (BCAN), and dibromoacetonitrile (DBAN)]; and (4) trichloronitromethane (TCNM). Since Taiwan EPA’s regulations specify a limit for HAA_5_ (below 60.0 μg/L; HAA_5_: the sum of MCAA, DCAA, TCAA, MBAA, and DBAA), but not for HAA_9_, we also considered SDS-HAA_5_ levels.

DBPs in water samples were extracted and concentrated using a liquid–liquid extraction (LLE) method (modified from USEPA methods 551.1 and 552.3). For THM_4_, HAN_4_ and TCNM, 30 mL of dechlorinated samples were placed in a 40 mL glass bottle, and 3 mL of solvent methyl tert-butyl ether (MTBE, Macron, Radnor, PA, USA) and 10 g of sodium sulfate salt (Macron) were added. After vigorous mixing, the solution was left to sit until the two phases were completely separated. The upper layer was transferred into a clean vial for GC-µECD analysis. For HAA_9_, 30 mL of dechlorinated samples were placed in a 40 mL glass bottle, and 3 mL of solvent methyl tert-butyl ether (MTBE, Macron, USA), 10 g of sodium sulfate salt (Macron) and 98% sulfuric acid were added. After vigorous mixing and phase separation, one milliliter of the upper layer was transferred into a clean vial with 1 mL of 10% (v/v) sulfuric acid in methanol solution. The vial was incubated in a 50 °C water bath for 2 h for derivatization. After the solution had cooled to room temperature, a mixture of 1 mL of MTBE and 3 mL of 15% sodium sulfate was used as a solvent to extract DBPs. Two mL of 15% sodium sulfate was then used to reverse extract HAA_9_ twice. Finally, the upper layer was transferred to 2 mL vials before the DPBs were analyzed using GC-µECD (6890N Gas Chromatograph, Agilent Technologies, Santa Clara, CA, USA) with a DB-1701 column (30 m × 0.250 mm, 0.25 µm film thickness, Agilent Technologies).

## 3. Results

### 3.1. DOC Removal

**Figure 2:**
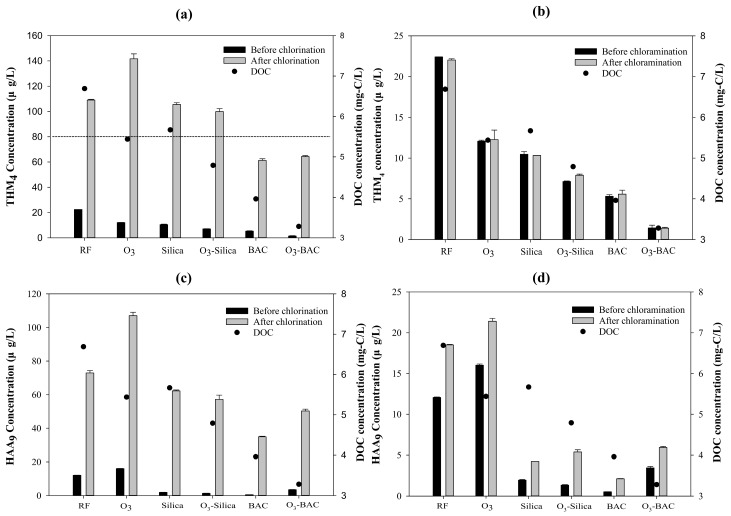
Simulated distribution system (SDS) tests: carbonaceous disinfection byproducts (DBPs). The DBPs measured were THM_4_, with chlorine (**a**) or chloramine (**b**) as the disinfectant, and HAA_9_, with chlorine (**c**) or chloramine (**d**) as the disinfectant. Effluent from rapid sand filtration (RF) was treated using ozonation and/or biofiltration processes. Solid black and gray bars respectively represent the concentrations of the disinfection byproducts before and 24 h after the indicated disinfectants had been added. Solid circles indicate the corresponding DOC concentrations. The broken line (panel a) indicates the Taiwan EPA’s regulatory limit for THM_4_. (O_3_: ozonation; Silica: silica sand biofiltration; BAC: biological activated-carbon biofiltration).

The DOC concentration of the RF was high (6.69 mg C/L), and all the treatment processes tested in this study reduced this concentration, particularly those that included BAC filtration (Figure 2). DOC concentrations decreased to 3.96 and 3.28 mg C/L for the BAC and O_3_–BAC treatments, respectively. Lower removal rates were observed for the O_3_, Silica, and O_3_–Silica processes, for which the final concentrations were 5.44, 5.67, and 4.79 mg C/L, respectively. As can be seen, for both the Silica and the BAC processes, the addition of the O_3_ treatment enhanced the removal of DOC. In summary, the removal efficiency was greatest for the O_3_–BAC treatment, which reduced DOC by 51%, followed by BAC (41%), O_3_–Silica (28%), O_3_ (19%), and Silica (15%).

### 3.2. Simulated Distribution System (SDS) Test of Rapid Sand Filtration Effluent (RF) Treated by Ozonation and/or Biofiltration

#### 3.2.1. SDS Tests: Trihalomethanes (THM_4_)

SDS-THM_4_ increased dramatically after chlorination (Figure 2a, gray bar for RF), indicating that substantial quantities of THM_4_ precursors remained in the RF effluent after the preceding water treatment processes. All selected treatments reduced SDS-THM_4_ concentrations relative to the RF control, except for ozonation alone (O_3_), which elevated the SDS-THM_4_ concentration from 109.1 μg/L (RF) to 141.7 μg/L (O_3_; 30% increase). The SDS-THM_4_ concentration was lowest after BAC filtration (64.3 μg/L; 44% reduction), although there was only a negligible difference between O_3_–BAC (61.3 μg/L; 41% reduction) and BAC. Silica sand biofiltration achieved only a slight reduction in SDS-THM_4_, and ozonation added to this treatment had no significant additional effect on the removal of THM_4_ precursors (Silica: 105.5 μg/L, a 3% reduction; O_3_–Silica: 99.9 μg/L, an 8% reduction). Importantly, the SDS-THM_4_ concentrations were below the Taiwan EPA’s regulatory limit (80 μg/L) after both the BAC and the O_3_–BAC treatments.

When chloramine was used as the disinfectant, the concentration of SDS-THM_4_ remained low for all the 24 h tests, and did not exceed 15 μg/L for any of the treatment processes (Figure 2b). The level of SDS-THM_4_ was lowest for the O_3_–BAC treatment (1.4 μg/L), followed by the BAC (5.6 μg/L), O_3_–Silica (7.9 μg/L), Silica (10.3 μg/L), and O_3_ (12.3 μg/L) treatments.

#### 3.2.2. SDS Tests: Haloacetic Acids (HAA_9_)

With respect to SDS-HAA_9_ concentrations, when chlorine was used as the disinfectant (Figure 2c), the concentrations were lower when the treatment processes included BAC filtration (BAC: 34.9 μg/L, a 52% reduction; O_3_–BAC: 50.3 μg/L, a 31% reduction), but were only slightly reduced by silica sand biofiltration (Silica: 62.3 μg/L; O_3_–Silica: 57.2 μg/L) relative to the RF control (72.9 μg/L). As for THM_4_, ozonation alone significantly increased the concentration of SDS-HAA_9_ (107 μg/L; 47% increase). Although there is no Taiwan EPA regulatory limit for HAA_9_, the 24 h SDS-HAA_5_ concentrations met the requirement (<60 μg/L) after the BAC, O_3_–BAC, and O_3_–Silica treatments (data not shown).

When chloramine was used as the disinfectant (Figure 2d), the SDS-HAA_9_ concentrations remained low over 24 h (<21.4 μg/L). The concentrations were reduced by the processes involving silica sand or BAC filtration, but once again, were higher for the treatment processes involving ozonation. For example, simulated HAA_9_ concentrations increased from 18.5 μg/L (RF) to 21.4 μg/L (O_3_), from 2.1 μg/L (BAC) to 5.9 μg/L (O_3_–BAC), and from 4.3 μg/L (Silica) to 5.4 μg/L (O_3_–Silica) after ozonation.

#### 3.2.3. SDS Tests: Haloacetonitriles (HAN_4_)

**Figure 3:**
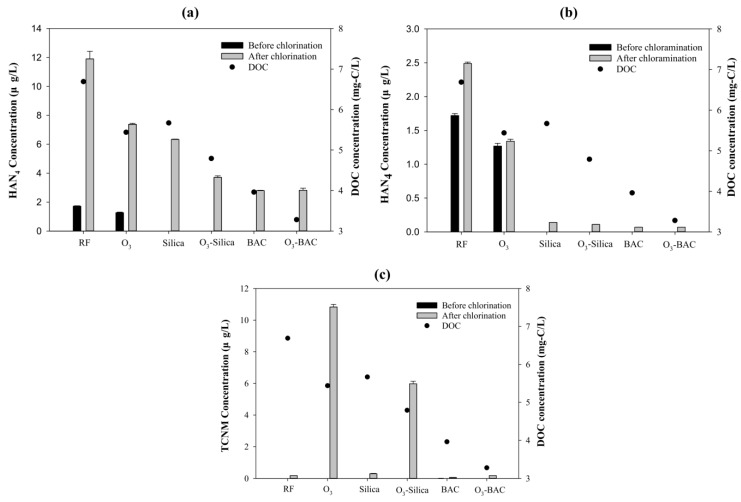
Simulated distribution system (SDS) tests: nitrogenous disinfection byproducts (DBPs). The DBPs measured were HAN_4_, with chlorine (**a**) or chloramine (**b**) as the disinfectant, and TCNM with chlorine as the disinfectant (**c**). Effluent from rapid sand filtration (RF) was treated by ozonation and/or biofiltration processes. Solid black and gray bars represent the concentrations of disinfection byproducts before and 24 h after adding indicated the disinfectants, respectively. Solid circles indicate DOC concentrations. (O_3_: ozonation; Silica: silica sand biofiltration; BAC: biological activated-carbon biofiltration).

All five of the treatments reduced the simulated levels of HAN_4_. The best SDS-HAN_4_ removal efficiency was obtained with BAC biofiltration (Figure 3a,b). For tests using chlorine as a disinfectant, the lowest SDS-HAN_4_ concentration was observed for the BAC and O_3_–BAC treatments (both 2.8 μg/L; 76% reduction), followed by the O_3_–Silica (3.7 μg/L), Silica (6.3 μg/L), and O_3_ (7.4 μg/L) treatments (Figure 3a). For the tests using chloramine as a disinfectant, biofiltration was again highly effective in reducing the simulated HAN_4_ levels, regardless of whether silica sand or BAC was used (<0.1 μg/L). However, unlike with THM_4_ and HAA_9_ species, the precursors of HAN_4_ were not generated by ozonation.

#### 3.2.4. SDS Tests: Trichloronitromethane (TCNM)

In contrast to the results obtained for the other DBPs, only small amounts of TCNM were detected after chlorination except the treatments involving ozonation. Ozonation significantly increased the yields of SDS-TCNM (Figure 3c): the concentrations after treatment with O_3_ (10.8 μg/L), O_3_–Silica (6.0 μg/L), and O_3_–BAC (0.2 μg/L) were much higher than those for the RF (0.2 μg/L), Silica (0.3 μg/L), and BAC (0.1 μg/L) treatments, respectively. The lowest SDS-TCNM concentration was obtained using BAC biofiltration (0.1 μg/L, an 11% reduction). In addition, BAC (0.2 μg/L) performed much better than Silica (6.0 μg/L) in removing O_3_-induced TCNM. In contrast to chlorination, TCNM yields after chloramination were generally below 0.13 μg/L (data not shown). Chloramine was thus more effective than chlorine in controlling the generation of TCNM during disinfection.

## 4. Discussion

### 4.1. Effect of Treatments on the Removal of DOC

After rapid sand filtration, the DOC level remained at 6.7 mg/L (only 54% DOC removal; Figure 2), indicating that the preceding conventional water treatment processes (coagulation, flocculation, and rapid sand filtration) were not effective in removing high concentrations of organic substances from source water. A large proportion of the organic matter in the source water was probably derived from cyanobacterial blooms, which would have occurred because of the excess nutrients discharged into the reservoir. This result is consistent with a previous study [22] that showed that extracellular and intracellular algal organic matter, especially the lower-molecular-weight fractions, was difficult to remove using conventional water treatment processes.

Ozonation followed by biofiltration (either Silica or BAC) reduced the DOC concentrations compared to biofiltration alone (Figure 2), implying that ozone treatments generate readily biodegradable organic carbon [16,17,18]. The ability of ozone to transform DOC has been documented in several studies. For example, ozonation can (1) convert hydrophobic natural organic matter (NOM) to hydrophilic NOM; (2) decompose high-molecular-weight fractions of NOM to compounds of lower molecular weight; and (3) preferentially attack double bonds and aromatic rings to produce small biodegradable molecules such as carboxylic acids and aldehydes [16,17,18,23,24].

In addition, a greater decrease in DOC was observed for biofiltration with activated carbon (O_3_–BAC: 51%) than with silica sand (O_3_–Silica: 28%). This is probably because activated carbon has a much higher surface area to which microbes can attach and grow, resulting in higher DOC utilization. Enhanced attachment of microorganisms has been demonstrated by observing higher biomass [25] and concentrations of ATP, proteins, and polysaccharides on the surface of granular activated carbon (GAC) [15]. The lower DOC removal observed in the Silica treatment was probably due to the lower surface area and roughness of silica sand, which makes it less suitable for microbes to attach to and develop biofilms. Since the BAC and silica columns were acclimated using local pond water (DOC 3–6 mg/L) for at least six months before the experiments, adsorption of organic substances by the filter media alone was less likely. The DOC removal could thus have resulted from direct biological utilization and/or from a bioregeneration mechanism (i.e., the regeneration of active adsorption sites on GAC via the microbial consumption of organic molecules on occupied sites) [26].

In summary, the sequential ozonation–BAC filtration process outperformed the other treatments in this study in terms of cyanobacterial DOC removal efficiency (51%). BAC filtration on its own also substantially reduced the amount of organic carbon (41%). 

### 4.2. Effect of Treatments on the Reduction of DBP Yields

Since DOC reduction is crucial in controlling DBP formation after disinfection, SDS-DPBs were also measured, to evaluate the treatment processes.

#### 4.2.1. Carbonaceous DBPs: THM_4_

Only the BAC and O_3_–BAC treatments both reduced SDS-THM_4_ levels to below 80 μg/L and maintained residual free chlorine in finished water in the range 0.2–1.0 mg/L (Figure 2a, Table 1). Combined ozonation and biofiltration has previously been used to control DOC levels and DBP formation [24,27,28]. In our study, biofiltration using activated carbon (BAC or O_3_–BAC) yielded the lowest SDS-THM_4_ concentrations (approximately 60 μg/L; 40% reduction), regardless of a preceding ozonation treatment. However, utilization of ozone can decrease DOC by an additional 10% (see Section 4.1). Removal of DOC from finished water can reduce the DBP-formation potential as well as lower the risk of microbial regrowth in water distribution systems. Nevertheless, the possibility of regrowth was minimized in our experiments, since (1) the readily biodegradable fraction of DOC (i.e., assimilable organic carbon) was used by microorganisms inhabiting the biofiltration columns, and (2) relatively high concentrations of residual disinfectants remained in the 24 h SDS tests. This implies that BAC on its own might be an effective and economical option if ozonation is not available. Only a slight reduction in SDS-THM_4_ concentration, relative to the RF control, was observed for the Silica and O_3_–Silica treatments. In terms of the removal of THM_4_ precursors, the biofiltration treatments using activated carbon were more effective than those utilizing silica sand. This was probably due to the higher biomass in the BAC columns.

In this study, ozonation on its own had a negative effect on the reduction of SDS-THM_4_ (Figure 2a). The effects of ozonation on the THM-formation potential (both positive and negative) have been reported in various studies, and appear to depend on the characteristics of the DOC. For example, for organic matter with a high humic content or high SUVA values (>2 L/mg/m), ozonation not only transforms it from hydrophobic fractions (more reactive to chlorine) into hydrophilic fractions (less reactive to chlorine), but also partially oxidizes both fractions, which reduces their reactivity towards disinfectants [23,29,30,31]. In contrast, for organic matter with low SUVA values, such as algal organic matter, ozonation increases the yield of C- and N-DBPs after subsequent chlorination or chloramination [8,15]. Furthermore, it may disrupt intact algal cells and thus increase DOC levels by releasing IOM, which is considered a source of DPB precursors [8,15]. Consistent with this, we observed an increase in THM_4_-formation potential after ozonation in the present study, probably because the ozonation of algae-derived DOC generates more THM_4_ precursors. In summary, both the BAC and O_3_–BAC treatments reduced SDS-THM_4_ below the regulated level, and the O_3_–BAC treatment also decreased DOC by an additional 10%. BAC alone was sufficient to reduce SDS-THM_4_. If ozonation is applied in water treatment processes, therefore, a subsequent BAC biofiltration is highly recommended, in order to remove the THM_4_ precursors generated by reactions between cyanobacterial organic matter and ozone.

In contrast to chlorination, during chloramination the concentrations of SDS-THM_4_ were very low (below 23 μg/L), which is in agreement with the results of other studies [15,30,31] (Figure 2b). Interestingly, the simulated THM_4_ concentrations before (Figure 2b, black bars) and after (gray bars) the addition of chloramine were similar, indicating that chloramination (with or without pre-ozonation) did not induce additional THM_4_ formation. The reasons for the inactivation of THM_4_ precursors against chloramination in this study are unclear. There are two likely explanations for this result: both (1) a low initial NH_2_Cl dose (≤1 mg Cl/L) and a short exposure time (24 h) in the SDS tests and (2) a pre-chlorination step before the coagulation/floatation processes in water treatment plants may inactivate the chloramine precursor sites in DOC.

#### 4.2.2. Carbonaceous DBPs: HAA_9_

When chlorine was used as the disinfectant, the reduction in SDS-HAA_9_ concentrations was similar to that of THM_4_ (Figure 2c): (1) BAC and O_3_–BAC resulted in the lowest concentrations of SDS-HAA_9_; (2) removal of HAA_9_ precursors was more effective by biofiltration with activated carbon (BAC or O_3_–BAC) than silica sand (Silica or O_3_–Silica), probably because of the attachment of a greater quantity of biomass to the surface of the activated carbon; and (3) ozonation of cyanobacteria-derived DOC increased SDS-HAA_9_ concentrations. It has been reported that algae-derived DOC, particularly IOM, is highly biodegradable [8]. BAC biofiltration can therefore be regarded as an efficient process that can be used in water treatment plants to reduce the level of HAA_9_ precursors when there are serious algal blooms in the source water.

We observed a negative effect in this study when ozone was used alone. The concentration of SDS-HAA_9_ was highest in the O_3_ treatment (107.0 μg/L). The BAC treatment on its own achieved a greater reduction in SDS-HAA_9_ concentration (52%) than the combination of pre-ozonation and BAC filtration (31%). The generation of HAA_9_ precursors by ozone-treated algal organic matter has been reported in previous studies [8,15], and furthermore, ozone can damage the cell envelope of cyanobacteria, releasing more IOM, which can later increase HAA_9_ yields; indeed, the IOM of several cyanobacterium species has been identified as an important source of HAA_9_ precursors [6,8,15].

We also considered HAA_5_ levels, because the Taiwan EPA has a regulatory limit for HAA_5_ but not for HAA_9_. The SDS-HAA_5_ levels in the untreated control (RF) were 65.1 μg/L, but all treatments except O_3_ on its own (86.7 μg/L) reduced the HAA_5_ concentration to below the regulatory limit, 60.0 μg/L (53.2, 44.6, 27.7, and 36.2 μg/L for the Silica, O_3_–Silica, BAC, and O_3_–BAC treatments, respectively). The fact that ozonation on its own yielded levels exceeding the regulatory limit suggests that a biofiltration process should be applied after ozone treatment.

The SDS tests revealed similar patterns for chlorination and chloramination, but with much lower simulated HAA_9_ concentrations for chloramination (Figure 2d). A large fraction of the HAA_9_ concentration originated from the pre-chlorination process (Figure 2d, solid black bars) and was removed by subsequent biofiltration. Only a small quantity of SDS-HAA_9_ was produced during chloramination, consistent with the results of a previous study [15]. These results indicate that chloramine is an excellent disinfectant for controlling the yield of SDS-HAA_9_.

#### 4.2.3. Nitrogenous DBPs: HANs

In the chlorination treatments, the greatest reduction of HAN_4_ precursors was observed in the BAC and O_3_–BAC treatments (both removed 76% of HAN precursors), followed by O_3_–Silica (69%), Silica alone (48%), and O_3_ alone (38%; Figure 3a). This suggests that biofiltration, particularly with activated carbon, can efficiently reduce the HAN_4_-formation potential during subsequent chlorination. In contrast to the results for SDS-THM_4_ and SDS-HAA_9_, ozonation on its own reduced the yield of HANs, despite opposite results (increase in DCAN yields) reported by Zhou et al. [15]. These authors showed that when algal organic matter was treated the formation of DCAN increased by approximately 10% after pre-ozonation and chlorination. They hypothesized that this may have been caused by a shift from high-molecular-weight organic substances (>100 kDa) to a low-molecular-weight fraction (<10 kDa), because the yield of DPBs was generally higher. In most cases, however, ozone is quite effective in suppressing the formation of HANs. For example, DHANs were found to have the highest sensitivity to ozone treatment during chlorination among a number of other DPBs (THMs, THAAs, and DHAAs) [30]. In addition, significant reductions in DCAN and BCAN concentrations after ozonation were reported by Chiang et al., who examined the same source water [19], and Yang et al. [32] showed that O_3_ pretreatment reduced the yield of HANs during subsequent chlorination. The results of this study thus suggest that a combination of BAC filtration and ozonation is highly effective in controlling the generation of HAN_4_ during chlorination.

As shown in Figure 3b, the SDS-HAN_4_ concentrations with chloramination were much lower than with chlorination. Biofiltration with either silica sand or activated carbon exhibited an excellent removal rate of HAN_4_ precursors (94–97%). Ozonation alone reduced HAN_4_ yields by 46% compared with the control. This result is in agreement with other studies showing that pre-ozonation and chloramination greatly suppress HAN_4_ concentrations [15,30,32,33]. 

In summary, BAC filtration (alone or in combination with pre-ozonation) was highly effective in reducing HAN_4_ concentrations in source water with high algae-derived organic matter concentrations. In contrast to the C-DBPs, ozonation on its own was effective in reducing HAN_4_ concentrations. Similar patterns of HAN_4_ removal by the treatments we tested were observed for chlorination and chloramination, but chloramine induced much lower SDS-HAN_4_ concentrations than chlorine.

#### 4.2.4. Nitrogenous DBPs: TCNM

Under chlorination, the TCNM concentration was lowest when BAC filtration was applied (Figure 3c). Ozonation had a strong negative effect on the reduction of TCNM precursors, consistent with other research [8,15,19,34,35]. The yield of TCNM after treatment with ozonation alone was 60 times higher than that obtained without any treatment (RF). For source water that has undergone large cyanobacterial blooms, therefore, the use of ozone by water treatment plants should be carefully evaluated, if TCNM generation is a concern. This is especially the case because amino acids and amino sugars that are common in algal cells have been identified as important TCNM precursors [34]. The results of the present study also show that ozone-generated TCNM precursors can be removed by subsequent biofiltration, using either silica sand (O_3_–Silica) or activated carbon (O_3_–BAC). However, the removal efficiency of TCNM precursors was much better with the BAC (98%) than the Silica (45%) treatment. Under chloramination, TCNM concentrations were significantly lower (<0.13 μg/L) than chlorinated TCNM concentrations (data not shown), suggesting that chloramine is also effective in controlling TCNM concentrations.

## 5. Conclusions

In summary, our 24 h SDS tests suggest that applying BAC biofiltration after rapid sand filtration is an effective and economical approach to reducing DBPs precursors. Ozonation before BAC filtration performed similarly with respect to reducing SDS-DPBs, and also achieved a 10% larger reduction in DOC. Ozonation following rapid sand filtration is not recommended without a subsequent biofiltration process, because it was found to elevate SDS-THM_4_, SDS-HAA_9_, and SDS-TCNM concentrations. The results of this study may provide useful information for water treatment plants that wish to reduce DBP yields, particularly when the source water has undergone extensive cyanobacterial blooms. A future pilot test using real pipe material would be helpful for confirming the laboratory results obtained here.

## Figures and Tables

**Table 1:** Residual disinfectant concentrations at the end of the simulated distribution system tests (24 h SDS tests) for the proposed treatment processes.

Treatment Process ^a^	Average Residual Chlorine at 24 h (mg Cl_2_/L)		
	NaOCl		NH_2_Cl
	Free Chlorine	Total Chlorine	Total Chlorine
RF	0.51	0.94	0.69
O_3_	0.54	0.92	0.65
Silica	0.67	1.09	0.58
O_3_–Silica	0.68	1.00	0.61
BAC	0.48	0.78	0.59
O_3_–BAC	0.52	0.73	0.66

SDS—simulated distribution system; ^a^ Treatment process: RF (effluent from rapid sand filtration); O_3_ (ozonation); Silica (silica sand biofiltration); O_3_–Silica (ozonation followed by silica sand biofiltration); BAC (biological activated-carbon filtration); O_3_–BAC (ozonation followed by biological activated-carbon filtration).
